# Neuropsychiatric Systemic Lupus Erythematosus

**DOI:** 10.2174/157015911796557984

**Published:** 2011-09

**Authors:** Alexandra Popescu, Amy H Kao

**Affiliations:** 1 Department of Neurology, Epilepsy Division, University of Pittsburgh School of Medicine, Pittsburgh, PA, USA; 2 Lupus Center of Excellence, Department of Medicine, Allegheny Singer Research Institute, West Penn Allegheny Health System, Pittsburgh, PA, USA

**Keywords:** SLE, neuropsychiatric lupus, immunosuppression, autoimmunity, autoantibody.

## Abstract

Neuropsychiatric systemic lupus erythematosus (NPSLE) is the least understood, yet perhaps the most prevalent manifestation of lupus.  The pathogenesis of NPSLE is multifactorial and involves various inflammatory cytokines, autoantibodies, and immune complexes resulting in vasculopathic, cytotoxic and autoantibody-mediated neuronal injury.  The management of NPSLE is multimodal and has not been subjected to rigorous study. Different treatment regimens include nonsteroidal anti-inflammatory drugs, anticoagulation, and immunosuppressives such as cyclophosphamide, azathioprine, mycophenolate mofetil, and methotrexate.  For refractory NPSLE, intravenous immunoglobulin (IVIG), plasmapheresis, and rituximab have been used.  Adjunctive symptomatic treatment complements these therapies by targeting mood disorders, psychosis, cognitive impairment, seizures or headaches.  Several new biological agents are being tested including Belimumab, a human monoclonal antibody that targets B lymphocyte stimulator.  This review focuses on the pathophysiology, treatment, and new potential therapies for neuropsychiatric manifestations of systemic lupus erythematosus.

## I. INTRODUCTION

An autoimmune disease is a disorder in which the misdirected immune system reacts against host antigens. The dysregulation of the immune system may precipitate dysfunction and damage of various organ systems, including the central and peripheral nervous systems. We are reviewing pathophysiology of neuropsychiatric involvement in systemic lupus erythematosus (SLE) and its therapy. Most of the clinical manifestations of disease are nonspecific complicating the diagnosis, clinical assessment and treatment planning.

## II. CLINiCAL FEATURES AND EPIDEMIOLOGY

SLE is an autoimmune disease that predominantly affects women of child-bearing age. In the United States, SLE is more prevalent among African Americans, Hispanics, and Asians compared to non-Hispanic Caucasians [1]. This heterogeneous autoimmune disease is characterized by the loss of tolerance to autoantigens and the development of immune complexes that deposit in tissues and cause systemic inflammation. SLE also involves a number of cytokine pathways, including B lymphocyte stimulator (BLys), which promotes B-cell survival and autoantibody production, type I interferon (IFN), which acts as immune adjuvant, and tumor necrosis factor, which contributes to organ inflammation. Type I IFNs are normally produced by plasmacytoid dendritic cells in response to viral infection. However, patients with SLE have an increased expression of type I IFN regulated genes due to the continuous production of IFN-α. Their dendritic cells activated by immune complexes containing endogenous nucleic acids are also induced to synthesize IFN that contributes to loss of tolerance and activation of autoreactive T and B cells with production of autoantibodies [2-4]. SLE patients have elevated serum levels of IFN-α, which correlate with both disease activity and severity [5, 6]. Based on this knowledge, many clinical trials targeting cytokines as potential therapies for SLE are underway. 

Neuropsychiatric lupus (NPSLE) is the least understood yet perhaps the most prevalent manifestations of lupus. It affects 14% to over 80% in adults [7-12] and 22% to 95% in children [11, 13-16] and can occur independently of active systemic disease and without serologic activity [17]. NSPLE is associated with increased morbidity and mortality [7-9, 18, 19]. In 1999, the American College of Rheumatology (ACR) established case definitions for 19 specific neuropsychiatric syndromes, dividing them into two broad categories: central and peripheral as shown in Table **1** [20]. In NPSLE, cognitive impairment is one of the most common manifestations, with a varying prevalence of 15% to 66% due to the differences in definition in the studies of mainly adult lupus patients [21-25]. Seizure and psychosis, however, are the only two NPSLE manifestations that comprise the neurologic component of the ACR classification criteria for SLE [26, 27]. It is estimated that 28% to 40% of adult NPSLE manifestations develop before or around the time of the diagnosis of SLE and 63% occur within the first year after diagnosis [9, 19, 28].

## III. PATHOPHYSIOLOGY AND PATHOGENESIS OF NEUROPSYCHIATRIC LUPUS

The pathogenesis of NPSLE is multifactorial and can involve various inflammatory cytokines, autoantibodies, and immune complexes resulting in vasculopathic, cytotoxic and autoantibody-mediated neuronal injury. The most common microscopic brain finding in SLE seems to be microvasculopathy although not specific, which may due to complement activation and antiphospholipid antibodies [29]. Post mortem histopathologic studies in people with SLE have demonstrated an array of pathologies including multifocal microinfarcts, gross infarcts, hemorrhage, cortical atrophy, ischemic demyelination, and patchy multiple sclerosis–like demyelination [30].

Disruption of the blood-brain barrier is integral to the neuropathology of SLE [31]. Endothelial cells responsible for maintenance of the blood-brain barrier may have altered function in the presence of elevated levels of ICAM-1, with subsequent normalization of function corresponding to both lower levels of ICAM-1 and disease remission [32]. Additionally, damage to the blood-brain barrier may be implicated in corticosteroid-induced psychiatric disorders [33]. Diamond and colleagues have generated abundant evidence from murine models, supporting the role of circulating anti-DNA autoantibodies mediating NPSLE *via *cross-reactivity with NR2 subunits of the anti-N-methyl-D-aspartate receptors during a breach of blood-brain barrier from inflammation or stress [34-37]. This cross-reactive excitatory neuronal death in the hippocampus was non-inflammatory by histopathologic examination and could be prevented by the administration of memantine, a NR2 glutamate receptor antagonist [36]. Similarly, epinephrine, a catecholamine, also breached the blood-brain barrier and caused selective neuronal loss in the lateral amygdala, leading to emotional disorder in the murine model [35]. These studies suggest that agents such as epinephrine can determine the region of brain that is made vulnerable to neurotoxic autoantibodies. 

While interruption of the function of the blood-brain barrier seems to play a role in the pathophysiology of NPSLE, there are several types of autoantibodies which are also associated with NPSLE. In addition to the aforementioned anti-NR2A glutamate receptor antibodies, intrathecal IgG production is often elevated during a central nervous system flare, and then normalizes with the resolution of the flare [38, 39]. This fluctuation in IgG levels is in contrast to the persistent abnormal immunologic indices seen during a remission of multiple sclerosis [40]. Moreover, approximately 25% to 40% of lupus patients have secondary antiphospholipid syndrome with clinical features of repeated episodes of arterial or venous thrombosis, recurrent spontaneous abortions, or thrombocytopenia in patients with antiphospholipid (aPL) antibodies [41-45]. Of note, patients with primary aPL syndrome do not fulfill the criteria for SLE, or other autoimmune disorders. APL antibodies target anionic phospholipids and protein-phospholipid complexes. Specifically, they can target negatively charged phosphatidyl glycerol, phosphatidyl serine, phosphatidyl acid, phosphatidyl inositol, and cardiolipin, in addition to the neutrally charged phosphatidyl ethanolamine, phosphatidyl choline, platelet activating factor, and sphingomyelin [46]. Using ELISA and lupus anticoagulant tests, antibodies binding cardiolipin in the presence of β2-glycoprotein-1 and antibodies binding β2-glycoprotein-1 and prothrombin can be detected. Studies have demonstrated aPL-mediated direct neuronal injury in the absence of ischemia [47-50]. Persistently elevated anticardiolipin antibodies are associated with greater cognitive impairment [23, 24]. In a cohort of 1000 patients followed over 10 years, Cervera and colleagues demonstrated an increased risk of thrombotic events, the most common being stroke which was observed in 11.8% of the cohort [51]. Among SLE patients with and without active disease both thrombotic events and cognitive impairment have been consistently linked to the presence of aPL, such as lupus anticoagulant and anticardiolipin antibodies [21, 52-54]. Additionally, transverse myelitis is highly associated with the presence of aPL [55]. Significant correlations between anticardiolipin IgG antibodies and a reduction in psychomotor speed, and between anticardiolipin IgA antibodies and a reduction in conceptual reasoning and executive ability have been found [21]. While the role of these autoantibodies awaits elucidation, they certainly can act as proxies for disease markers which may aid in the diagnosis of NPSLE.

The pathophysiologic role of autoantibodies in NPSLE is under ongoing investigation. In particular, the previously mentioned anti-NR2 glutamate receptor antibodies are seen in 25% to 30% of patients with SLE and may also play a role in cognitive dysfunction and psychiatric disease [56, 57]. These antibodies are anti-DNA antibodies that cross-react with the NR2 glutamate receptor and mediate excitatory apoptotic cell death of neurons *in vitro* and *in vivo* [37]. The anti-ribosomal P antibodies, another potential contributors to the pathogenesis of NPSLE, have not been implicated in coexisting cognitive impairment [58, 59]. Some studies suggested an association between serum anti-ribosomal P antibodies and NPSLE syndromes of psychosis and depression [59-63]. An international meta-analysis of 1,537 patients with SLE found the negligible value of anti-ribosomal P antibodies for the diagnosis of NPSLE or for specific NPSLE manifestations [64]. The potential role of anti-ribosomal P antibodies in the pathogenesis of NPSLE remains controversial. A cellular protein found strictly in neurons and essential to the cytoskeletal integrity is MAP-2. In a study of 100 patients with SLE and 74 patients with various neurologic disorders, more SLE patients comparing to neurologic injury/disease control patients have presence of anti-MAP-2 antibodies (17% *vs*. 4%, p=0.028 ) [65]. More specifically, 76.5% of NPSLE had presence of serum anti-MAP-2 antibodies. Using immunoproteomics, MAP-2B proteins were found to be preferentially recognized by sera from NPSLE patients, which further supports this association between the anti-MAP-2 antibodies and NPSLE [66]. The importance of autoantibodies is still under active investigation and many of the observations are based only on association. 

Other possible intrathecal markers for NPSLE include matrix metalloproteinase-9 (MMP-9) and plasminogen activator inhibitor 1 (PAI-1). MMP-9 is secreted by cells found in the walls of the vasculature, including macrophages, T lymphocytes, endothelial cells, and smooth muscle [67]. Its primary function is to enhance T cell migration through connective tissue. Significantly elevated intrathecal levels of MMP-9 are found in all patients with SLE comparing to non-SLE patients and specifically, with more elevation in NPSLE patients when compared with SLE patients without NPSLE [68]. Furthermore, CSF levels of IL-6 and IL-8, which are found to be elevated in NPSLE, are both significantly correlated with MMP-9 levels. Similarly, intrathecal levels of PAI-1 have been found to be significantly elevated in patients with NPSLE comparing to those without NPSLE and healthy controls [69]. The intrathecal levels of PAI-1 also correlated with CSF levels of proinflammatory cytokines, IL-6 and IL-8, in addition to association with neuronal damage markers, glial fibrillary acidic protein and neurofilament triplet protein. The association between neuronal injury and intrathecal homeostasis imbalance contributed by the release of PAI-1 suggests a potential therapeutic role of anticoagulation in patients with NPSLE even in the absence of the antiphospholipid syndrome.

## IV. NEUROIMAGING MODALITIES

Localizing the areas of the CNS associated with neuropsychiatric symptoms in SLE continues to be elucidated with brain imaging studies, though these modalities are not without limitations. While focal neurologic symptoms of NPSLE correlate with conventional structural magnetic resonance imaging (MRI) abnormalities, abnormalities reflecting altered perfusion or neurometabolite changes in NPSLE can be demonstrated by functional imaging techniques even in the absence of morphological lesions detectable by conventional MRI. Cortical atrophy, ventricular dilation, diffuse white matter, and gross infarctions are common [70-74]. Using structural MRI, 40%–80% of abnormalities in NPSLE are multiple discrete lesions concentrated in periventricular and subcortical white matter [75]. These can also be seen in SLE patients without past or active neuropsychiatric lupus [76]. Hippocampal atrophy correlates with disease duration, total corticosteroid dose, and repeat CNS events in patients with SLE [77]. The presence of hyperintense white matter lesions in SLE is associated with age, total corticosteroid dose received and Systemic Lupus International Collaborating Clinics (SLICC) Damage Index scores [78]. Furthermore, predictors for development of new or worsening of existing white matter lesions include past CNS involvement, elevated titers of aPL antibodies, SLICC Damage Index scores and higher dose of total corticosteroid dose [78].

Metabolic neuroimaging (positron emission tomography/PET, MR spectroscopy) and perfusion imaging (single photon emission computer tomography/SPECT) can detect abnormalities in patients who present exclusively with psychiatric manifestations, but otherwise have normal MRI studies. In fact, normal structural MRI studies do not exclude active NPSLE as fluorodeoxyglucose-PET (FDG-PET) imaging can demonstrate parieto-occipital white matter hypometabolism in 60% to 80% of patients [11, 79-81]. During acute NPSLE, FDG-PET reveals white matter abnormalities in the prefrontal, anterior cingulate, and inferior parietal regions [11, 82, 83]. Such abnormalities rarely include temporal regions [79]. Consistent with the vasculopathic nature of NPSLE, SPECT and proton MR spectroscopy (^1^H-MRS) studies suggest that cerebral atrophy and cognitive impairment in SLE patients may be related to chronic diffuse cerebral ischemia [17, 76, 84]. MRS is an MR technique that determines the biochemical composition or neurometabolites of brain tissues noninvasively. Reduced *N*-acetylaspartate (NAA) levels may indicate neuronal injury or death whereas increased choline (Cho) levels have been associated with gliosis and membrane breakdown. ^1^H-MRS shows neurometabolic abnormalities during both active and quiescent periods of NPSLE, most probably related to neuronal injury or loss and demyelination [85, 86] More specifically, SLE patients with moderate or severe cognitive dysfunction had significantly higher Cho/creatine (cr) as determined by ^1^H-MRS compared to those with mild or no cognitive dysfunction, and SLE patients with active disease had low NAA/cr that returned to normal range after disease remission. Conversely, patients with active SLE during follow-up developed significant reduction in NAA/cr [86]. Furthermore, in a small study with follow-up after 4-6 years from baseline evaluation, all new structural MRI lesions were anatomically associated with the previously detected abnormal areas identified by SPECT and ^1^H-MRS [87]. This study is confirmed by another group which also found neurometabolic changes, specifically increased Cho/cr ratio, in normal appearing white matter may predict future appearance of hyperintense white matter lesions in SLE [88]. Lesions detected by MRI have been shown to correlate with cognitive impairment measured by neuropsychological testing in 72% of SLE patients (Kappa statistics for agreement=0.42, p=0.005) [89]. Finally, a recent study demonstrated the close postmortem histopathologic association of premortem neurometabolite abnormalities in NPSLE [90]. This was a prospective cohort study of 168 patients with SLE who had ^1^H-MRS at baseline, during lupus flare, and at 3 years after enrollment. Correlation of histopathology and imaging showed that: 1) elevated Cho levels are independently associated with gliosis, vasculopathy and edema; 2) reduced NAA levels are associated with reduced neuronal-axonal density; and 3) presence of lactate correlates with necrosis, microhemorrhage, and edema [90]. 

Functional MRI (fMRI) is another neuroimaging technique that has been used to assess for cognitive function in SLE. Formal neuropsychological testing and fMRI using 3 paradigms (continuous performance task, N-back task and verb generation) were assessed in a pilot study of 10 patients with childhood-onset SLE [91]. Compared to the control group, the SLE group demonstrated significantly increased activation of brain areas involved in the 3 paradigms. More importantly, in the absence of active stimuli, SLE patients consistently undersuppressed activity in the expected brain area. These findings support the notion that the imbalance between active and inhibitory responses to stimuli in childhood-onset SLE may be associated with abnormalities in white matter connectivity resulting in neuronal network dysfunction.

Neuroimaging modalities have greatly advanced the understanding of NPSLE, which appears to be caused by acute and chronic brain injury caused by SLE and are promising approaches to elucidate the areas and mechanisms involved in NPSLE and specific manifestation as in cognitive dysfunction. 

## V. TREATMENT

The management of patients with NPSLE is multimodal and continues to be a major therapeutic challenge due to the broad spectrum of the NPSLE manifestations and limitations in diagnostic testing. Currently, only three medications are FDA-approved for treatment of SLE in the United States: glucocorticoids, aspirin, and hydroxychloroquine. Glucocorticoids are one of the primary therapeutics in the management of NPSLE. Treatment choices tend to vary and are tailored to individual patient’s clinical presentation, disease severity, and potential pathogenic mechanism. For example, thrombosis with the presence of antiphospholipid antibodies would indicate the need for anticoagulation. Medication use can range from nonsteroidal anti-inflammatory drugs for symptomatic relief, anticoagulation for thrombotic diseases, to the immunosuppressives for inflammation such as cyclophosphamide, azathioprine, mycophenolate mofetil, and methotrexate [92]. Evidence for the efficacy of these therapies is limited to uncontrolled clinical trials and anecdotal experience [93]. Adjunctive symptomatic treatment complements these therapies by targeting mood disorders, psychosis, cognitive impairment, seizures or headaches. Mild NPSLE may only need symptomatic treatment. Treatment strategies should include identification and treatment of any secondary causes of CNS dysfunction such as infection, increased intracranial pressure, medication side effects, or primary psychiatric disorders. 

Glucocorticoids (e.g. prednisone, methylprednisolone) are one of the three FDA-approved drugs for the treatment of SLE; currently in the United States, as many as 90% of SLE patients are treated with glucocorticoids [11]. The treatment with glucocorticoids is widely used in the management of seizures, refractory headache, chorea, transverse myelitis and other CNS manifestations of SLE. Glucocorticoids are used in high doses orally (1-2 mg/kg by mouth daily), or intravenously (usually 1 gram daily for 3 days, followed by daily high-dose oral glucocorticoids) for acute and severe flares. Many patients require chronic immunosuppressive therapy in order to suppress SLE disease activity. Treatment with glucocorticoids carries long-term morbidity and numerous well-known side effects including dyslipidemia, diabetes, hypertension, accelerated cardiovascular disease, and increased susceptibility to infection and osteoporosis. In addition, these drugs can induce adverse effects on cognitive functioning and psychiatric symptoms [22, 94]. Therefore, in order to avoid these potential side effects, patients are administered the lowest possible dose of the glucocorticoids. 

Antimalarial drugs, specifically hydroxychloroquine, have suspected immunomodulatory properties that may also provide lipid-lowering and antiplatelet effects, thus preventing thromboembolic events [95]. Hydroxychloroquine can be used as maintenance therapy to prevent disease flare and may also improve fatigue and possibly cognitive dysfunction. Hydroxychloroquine is considered the safest of the antimalarials and can be continued during pregnancy. The initial dose is 200 mg daily with a maintenance dosage of 200 to 400mg daily but less than 6.5 mg/kg ideal body weight. The main side effects include irreversible retinopathy and glucose-6-phosphate dehydrogenase-induced hemolysis. Toxic myopathy can be seen in antimalarial use with the presence of curvilinear bodies by electron microscopy in muscle biopsy specimens [96].

Anticoagulation is the mainstay of therapy with or without immunosuppressives in NPSLE related to thrombosis and the presence of aPL antibodies. Furthermore, anticoagulation may also be used to treat refractory headache in patients with aPL antibodies. Antiplatelet agents, especially aspirin, have also been prescribed in patients with aPL antibodies but without a history of thrombosis. Stratification of the risks of thrombosis, smoking cessation and the use of protective medications such as aspirin and antimalarials are important elements of thrombosis prevention in SLE.

Cyclophosphamide is an immunosuppressive and cytotoxic alkylating agent used in organ-threatening disease in SLE and other autoimmune disorders. Severe NPSLE manifestations, mainly CNS involvement like cerebrovascular disease due to inflammation and transverse myelitis, has been treated with cyclophosphamide. There are different regimens of cyclophosphamide, which are mainly studied for treatment of lupus nephritis [97]. Cyclophosphamide can be given as monthly intravenous (500–1000 mg/m^2^) doses for a 6-month induction period followed by quarterly maintenance doses for a period of 2 years [55, 98]; however, studies have shown that immunosuppressives with less toxicity can be used for maintenance. Monthly intravenous cyclophosphamide was found to be superior to methylprednisolone in a small, randomized, clinical trial in controlling refractory seizures, peripheral and cranial neuropathy, and optic neuritis with a similar incidence of new infections [99]. In the Euro-Lupus Nephritis trial, lower dosage of intravenous cyclophosphamide at 500 mg has been given biweekly for 3 months for treatment of proliferative lupus nephritis. This study found comparable clinical results of this low-dose intravenous cyclophosphamide (cumulative dose of 3 gram) followed by azathioprine and the high-dose group (6 monthly pulse cyclophosphamide followed by 2 quarterly pulses) [100]. The most important side effects of cyclophosphamide include susceptibility to a variety of opportunistic infections, hemorrhagic cystitis from acrolein (toxic metabolite), increased risk of malignancy (non-Hodgkin lymphoma, leukemia, and transitional cell carcinoma of the bladder), and potential ovarian or testicular failure [101]. Due to the toxicity of cyclophosphamide, the current view is that cyclophosphamide should be used for shorter duration with a lower dose if possible and not be continued for maintenance therapy. Immunosuppressives with better side effect profile, such as mycophenolate mofetil (MMF), have been used frequently for maintenance and even initial treatment of NPSLE manifestations.

Other agents such as azathioprine and methotrexate have been used in systemic lupus erythematosus to treat a variety of manifestations or have been used as glucocorticoid-sparing agents, but controlled trials are lacking [102]. Azathioprine, methotrexate, and MMF are antimetabolites, which inhibit de novo synthesis of purine and/or pyrimidine [103]. Azathioprine is a prodrug that is rapidly and almost completely converted to 6-mercaptopurine (6-MP) and methylnitroimidazole. Further metabolism of 6-MP inhibits several enzymes of purine synthesis. Methotrexate and its polyglutamate derivatives suppress inflammatory response through release of adenosine, suppress immune response by inducing the apoptosis of activated T lymphocytes and inhibiting the synthesis of both purines and pyrimidines. MMF is a prodrug of mycophenolic acid (MPA) and an inhibitor of inosine-5’-monophosphate dehydrogenase. MPA depletes guanosine nucleotides preferentially in T and B lymphocytes and inhibits their proliferation, thereby suppressing cell-mediated immune response and antibody formation. MPA also inhibits the expression of adhesion molecules and the recruitment of immune cells (lymphocytes and monocytes) into sites of inflammation. MMF has been used to reduce acute and chronic rejection in allograft recipients. MMF showed a significantly higher remission rate than intravenous cyclophosphamide in renal lupus [104]. However, in a large international randomized controlled trial, MMF (target dosage 3 grams/day orally) and intravenous cyclophosphamide (0.5 to 1 gram/meter^2^ in monthly pulses) have similar efficacy to short-term induction therapy (24 weeks) for active lupus nephritis [105]. Of note, more Black and Hispanic patients responded to MMF than intravenous cyclophosphamide [106]. MMF has also shown to be efficacious in non-SLE patients with multiple sclerosis and optic neuritis by reducing the relapse rate [107, 108]. These noncytotoxic immunosuppressives are more commonly used in NPSLE patients with peripheral nervous system involvement such as peripheral and cranial neuropathy. Furthermore, MMF may be used as maintenance therapy after initial treatment with cyclophosphamide in patients with severe NPSLE.

Intravenous immunoglobulin (IVIG), plasmapheresis, and rituximab have been used in central nervous system manifestations unresponsive to glucocorticoid therapy and/or cytotoxic therapy. IVIG has immunomodulatory effects by interaction with the anti-idiotypic network, interference with the complement and cytokines’ network, cytolysis of target cells through complement or antibody-dependent cell-mediated cytotoxicity and induction of apoptosis of target cells through Fc receptors, neutralization of pathogenic antibodies and modulation of the activation and co-stimulatory molecules affecting differentiation of T cells, B cells, and dendritic cells [109]. IVIG also suppresses the expansion of auto-reactive B lymphocytes through signaling of the FcgRIIB, idiotype-mediated inhibition of B cell receptors and neutralization of cytokines such as the B cell survival factors. IVIG has been given as 2 gm/kg with divided doses over two to five days to treat thrombocytopenia, renal disease, central nervous system manifestations, and pregnancy loss associated with the presence of antiphospholipid antibodies [110]. Plasmapheresis, administered four to six sessions over one to two weeks, may be another option for the treatment of NPSLE manifestations [92]. The rationale of plasma exchange is based on the rapid removal of circulating pathogenic autoantibodies, immunoglobulins, immune complexes, and toxins. One study showed a significant elevation of peripheral CD4+ CD25 ^high^ FoxP3^+^ suppressor T cells in SLE patients treated with repeated plasmapheresis resulting in significant clinical improvements, thereby suggesting the increase in regulatory T cells may be one of the reasons of the beneficial effects of plasmapheresis in SLE patients [111]. Rituximab, chimeric anti-CD20 monoclonal antibodies that deplete CD20+ B cell, has shown to be efficacious in treatment of refractory SLE in case reports including those with transverse myelitis and CNS vasculitis [112, 113].

### Adjunctive Medications Commonly Used for Symptomatic Treatment

NPSLE manifestations, such as seizures and headaches, should also be treated with a combination of symptomatic and immune-modulating therapy. Seizures are managed with standard anticonvulsants. Although these medications have been implicated in drug-induced lupus, they do not appear to alter idiopathic disease. The nonsteroidal anti-inflammatory drugs (NSAIDs) suppress mild acute inflammation alleviating musculoskeletal manifestations, fatigue and fever. NSAIDs can alleviate migraine headache frequently seen in patients with SLE. However, chronic NSAIDs use can also aggravate headache. They may be administered alone or given in combination with glucocorticoids, allowing a decrease in glucocorticoid dosage. There are numerous options when choosing NSAIDs and effectiveness varies widely, therefore doses should be individualized. The adverse effects of NSAIDs include nonsteroidal-induced nephropathy, gastrointestinal bleeding, and ibuprofen-induced aseptic meningitis [92]. Psychotropic medications (antidepressants, anxiolytics, and atypical antipsychotics) may have an important adjunctive role in SLE patients with affective disorder or psychosis. There is no standardized treatment in lupus psychosis. The treatment for acute psychosis includes a combination of antipsychotic medications as symptomatic treatment and glucocorticoids to control underlying disease activity [114, 115]. Steroid-induced psychosis can be seen in patients already on this medication. A temporal relationship between the initiation of glucorticoids and psychiatric events, and the resolution of psychiatric symptoms after reduction of glucorticoids is the main clinical feature in diagnosis of steroid-induced psychosis. Pharmacologic treatment aimed at cognitive enhancement has not been studied in NPSLE. However, the regular use of aspirin in older SLE patients with diabetes was associated with improved cognitive function in the SALUD study [116]. On the other hand, consistent glucocorticoid use, which may be a surrogate of more active or severe disease, is associated with decline in cognitive function.

Nonpharmacological approaches, like cognitive rehabilitation programs or psychological group intervention may be important in SLE patients with psychiatric disorders such as depression, anxiety or cognitive dysfunction with impaired attention, concentration and memory [117]. The heterogeneity of the neuropsychological manifestations and the affected cognitive domains has led to a paucity of controlled clinical trials for cognitive rehabilitation of SLE patients. Thus, the current therapeutic approach is not based on empirical findings, but on clinical experience and small clinical studies.

## VI. FUTURE PERSPECTIVES

Although the above therapeutics may demonstrate significant benefits for NPSLE patients even with severe and otherwise refractory disease, there is unfortunately no cure for SLE. A variety of new biological agents for treatment of SLE (non-NPSLE) are being tested in randomized clinical trials. Belimumab, a human monoclonal antibody that targets B lymphocyte stimulator (BLys), is the first successful late-stage (Phase 3) trial for treatment of SLE, which showed a clinically and statistically significant reduction in SLE disease activity and flare rates, steroid use, and prolonged time-to-first lupus flare in patients with seropositive SLE or having positive antinuclear antibodies (ANA≥1:80 and/or anti-double-stranded DNA ≥30 IU/mL) [118]. The second belimumab study also showed reduction in SLE disease activity and severe lupus flare rates but no improvement in physician global assessment or reduction in steroid use as seen in the BLISS-52 study [119]. The adverse events and infection rates were comparable in the belimumab and placebo groups. Gene therapy and stem cell transplantation are also being investigated as novel therapies for SLE. However, due to the vast heterogeneity of the NPSLE manifestations, the treatment selection will need to be individualized based on both NPSLE and non-NPSLE clinical manifestations. Prospective multicenter studies are greatly needed to improve our knowledge and to help establish guidelines for the treatment of NPSLE manifestations. 

## Figures and Tables

**Table 1:** Neuropsychiatric Syndromes of Systemic Lupus Erythematosus [11]

**Central Nervous System**
Aseptic meningitis
Cerebrovascular disease
Cognitive dysfunction
Headache
Movement disorder (Chorea)
Seizures
Acute confusional state
Anxiety disorder
Mood disorder
Psychosis
Demyelinating syndrome
Myelopathy (transverse myelitis)
**Peripheral Nervous System**
Autonomic disorder
Mononeuropathy
Cranial neuropathy
Plexopathy
Polyneuropathy
Acute inflammatory demyelinating polyradiculoneuropathy (Guillain-Barré syndrome)
Myasthenia gravis
